# A novel CAR-T cell product targeting CD74 is an effective therapeutic approach in preclinical mantle cell lymphoma models

**DOI:** 10.1186/s40164-023-00437-8

**Published:** 2023-09-22

**Authors:** Wing Keung Chan, Jessica Williams, Kinnari Sorathia, Betsy Pray, Kaled Abusaleh, Zehua Bian, Archisha Sharma, Ian Hout, Shamama Nishat, Walter Hanel, Shelby L. Sloan, Aneeq Yasin, Nathan Denlinger, Xiaoli Zhang, Natarajan Muthusamy, Sumithira Vasu, Marcos de Lima, Yiping Yang, Robert Baiocchi, Lapo Alinari

**Affiliations:** 1 Department of Internal Medicine, Division of Hematology, College of Medicine, The Ohio State University, 400 W. 12th Ave, 481D Wiseman Hall, Columbus, OH 43210 USA; 2 Department of Biomedical Informatics/Center for Biostatistics, The Ohio State University, Columbus, OH 43210 USA

**Keywords:** Mantle cell lymphoma, CD74, Chimeric antigen receptor

## Abstract

**Background:**

Mantle cell lymphoma (MCL) is a rare B-cell non-Hodgkin lymphoma subtype which remains incurable despite multimodal approach including chemoimmunotherapy followed by stem cell transplant, targeted approaches such as the BTK inhibitor ibrutinib, and CD19 chimeric antigen receptor (CAR) T cells. CD74 is a nonpolymorphic type II integral membrane glycoprotein identified as an MHC class II chaperone and a receptor for macrophage migration inhibitory factor. Our group previously reported on CD74's abundant expression in MCL and its ability to increase via pharmacological inhibition of autophagosomal degradation. Milatuzumab, a fully humanized anti-CD74 monoclonal antibody, demonstrated significant activity in preclinical lymphoma models but failed to provide meaningful benefits in clinical trials mainly due to its short half-life. We hypothesized that targeting CD74 using a CAR-T cell would provide potent and durable anti-MCL activity.

**Methods:**

We engineered a second generation anti-CD74 CAR with 4-1BB and CD3ζ signaling domains (74bbz). Through in silico and rational mutagenesis on the scFV domain, the 74bbz CAR was functionally optimized for superior antigen binding affinity, proliferative signaling, and cytotoxic activity against MCL cells in vitro and in vivo.

**Results:**

Functionally optimized 74bbz CAR-T cells (clone 42105) induced significant killing of MCL cell lines, and primary MCL patient samples including one relapse after commercial CD19 CAR-T cell therapy with direct correlation between antigen density and cytotoxicity. It significantly prolonged the survival of an animal model established in NOD-SCIDγc^−/−^ (NSG) mice engrafted with a human MCL cell line Mino subcutaneously compared to controls. Finally, while CD74 is also expressed on normal immune cell subsets, treatment with 74bbz CAR-T cells resulted in minimal cytotoxicity against these cells both in vitro and in vivo.

**Conclusions:**

Targeting CD74 with 74bbz CAR-T cells represents a new cell therapy to provide a potent and durable and anti-MCL activity.

**Supplementary Information:**

The online version contains supplementary material available at 10.1186/s40164-023-00437-8.

## Background

Mantle cell lymphoma (MCL) is an incurable subtype of B-cell Non-Hodgkin’s lymphoma (NHL) characterized by an increasing incidence over the past two decades [1]. MCL is conventionally classified into 3 morphological variants: classic, blastoid, and pleomorphic, with the last 2 being considered more aggressive and associated with poor prognosis. Immunophenotypically, MCL lymphoma cells are positive for pan-B cell markers such as CD19 and CD20 and are usually CD5+, SOX11+, FMC7+, CD10−, and CD23− though variations in some subsets of patients have been reported. Molecularly, MCL is characterized by the translocation (11;14) which results in cyclin D1 overexpression and cell cycle dysregulation [2, 3]. In addition, secondary chromosomal alterations targeting genes involved in the regulation of cell cycle, DNA damage response, and cell survival pathways involving *P53*, *BIM*, *RB1*, and *ATM*. Amplification of *BCL2* and *c-MYC* are frequently present in this disease [4].

Younger MCL patients are treated with intensive chemo-immunotherapy regimens followed by autologous stem cell transplant consolidation [3, 5–7]. However, the majority of older patients are treated with less intensive approaches. Outside allogeneic stem cell transplant, treatment of patients with relapsed or refractory MCL is typically palliative and primarily includes covalent or non-covalent Bruton Tyrosine Kinase (BTK) inhibitors [8], lenalidomide, or Bcl-2 antagonists [9]. Though the disease responds to initial treatment, relapse inevitably occurs and prognosis is overall poor. Thus, novel treatment approaches for this disease are needed. In 2020, the first and only anti-CD19 CAR-T therapy (Tecartus KTE-X19) was approved for the treatment of relapsed and refactory MCL. This approval was based on a phase II clinical trial conducted across multiple centers enrolling 74 patients with a history of relapsed or refractory MCL who had previously been treated with ibrutinib [10]. The overall response rate was 91% with 68% of the 68 treated patients achieving a complete remission. However, after a median follow-up of 35.6 months, the progression free survival (PFS) and overall survival (OS) were 25.8 and 46.6 months, respectively, suggesting that a significant portion of these patients will need additional treatment [11].

CD74 is a nonpolymorphic type II transmembrane glycoprotein with its N-terminus domain facing the cytosol that functions as an MHC class II chaperone promoting exogenous antigen processing and presentation [12]. In the endoplasmic reticulum (ER), a CD74 trimer binds to three MHCII alpha–beta dimers forming a nonameric structure, which then egresses the ER and translocates to the cell surface [13]. Interestingly, CD74 has been previously shown to specifically promote normal B cell proliferation and survival by activating downstream pathways such as NF-kB and B-cell receptor/PI3K/Akt signaling [14]. CD74 also functions as the receptor for the macrophage migration inhibitory factor (MIF), a proinflammatory cytokine secreted by various immune cell subsets [12]. It is known that CD74 is produced in molar excess in comparison to MHC class II, which results in its abundant free-loaded expression on the cell surface. CD74 is degraded through a proteolytic mechanism initiated by the cytoplasmic cleavage of its N-terminus domain by Signal Peptide Peptidase-Like 2A (SPPL2a). Consistent with its role in antigen presentation, CD74 is expressed on professional antigen presenting cells such as macrophages, dendritic cells, and B cells, as well as epithelial cells under inflammatory conditions [15]. Importantly, CD74 is expressed at much higher levels in a variety of hematologic malignancies, such as B and T cell lymphomas, multiple myeloma (MM), and chronic lymphocytic leukemia (CLL), in addition to solid tumors of the gastrointestinal tract [15–18]. We have previously reported the abundant expression of CD74 in MCL [19–21] and showed this to be a viable therapeutic target in this disease using milatuzumab, a naked fully humanized monoclonal antibody targeting CD74 [22]. Despite the attractive properties of CD74 as a therapeutic target and the significant activity of milatuzumab in preclinical MCL models, milatuzumab showed modest clinical activity, primarily due to rapid internalization of the antibody-antigen complex, resulting in a half-life of about 2 h. In a phase I clinical trial conducted in 22 patients with relapsed/refractory B-cell NHL, milatuzumab was overall well tolerated with the most common toxicities being infusion reaction and cytopenias but demonstrated very limited clinical activity with no objective response reported [23]. In a phase I–II clinical trial conducted in 8 patients with relapsed/refractory CLL, milatuzumab was overall well tolerated with improvement in performance status observed in most patients and a modest response in hematological parameters but with no patients meeting the iwCLL criteria for partial or complete response [18].

To overcome the limitations of milatuzumab, we developed and functionally optimized an anti-CD74 CAR-T cell product, called 42105-74bbz with 4-1BB + CD3ζ as co-stimulatory domains. We found that 42105-74bbz CAR-T cells exhibited cytotoxicity against both MCL cell lines and primary MCL patient samples, and significantly prolonged the survival in a preclinical MCL mouse model with limited off-tumor effect on normal immune cell subsets.

## Methods

### CD74 CAR-T vector construction and expression

A second-generation CAR was designed, where single chain variable fragments (scFV) of the mouse anti-human CD74 was joined with 4-1BB and CD3ζ chain via the CD8 hinge region and transmembrane domain. A CMV promoter was added in front of the entire sequence for constitutive expression. The sequence was then codon-optimized, synthesized (Twist Bioscience, OR), and subcloned into a lentiviral vector pCDH (SBI Bioscience, CA). Lentiviral particles were made by transfecting Lenti-X 293T cells (Clontech, Takara Bio USA Inc, CA) with the CAR construct and packaging plasmids including PsPax2, (Addgene #12260) and pMD2.G (Addgene #12259), via Lipofectamine 2000 (Invitrogen, MA). The viral supernatant was harvested 48 h post-transfection, passed through a 0.22 um filter, and then snap-frozen at − 80 °C until it was used.

### In silico scFV optimization and mutagenesis

Amino acid paratope in the regions of complementarity-determining region (CDR)1, CDR2, and CDR3 was first identified by ProABC-2. The scFV was then in silico reconstructed as PDB by ABodyBuilder2 [24] and prepared for antigen docking (CD74, PDB# 1IIE). The scFV was then docked to the antigen using HADDOCK 2.4 [25]. To refine the docking model, alanine scanning on the amino acid residues on each CDR was performed. Among all model cluster candidates, the one with the lowest HADDOCK score was picked for computational mutagenesis study (Additional file 1: Figure S1). The binding residue candidates were substituted and docked against CD74 antigen with all possible 20 amino acids. By using Eris molecular suite [26], mutations were introduced in the scFV regions on those binding residues. The estimated free energies of mutant conformations were compared with the wild-type and lead mutants were selected for site-directed mutagenesis and downstream functional assays. Mutagenesis on the CDR regions were performed by GeneMorph II EZClone domain mutagenesis kit (Agilent, CA) according to the manufacturer’s instructions. Three CAR constructs with anti-CD74 scFV (named 543, 553, and 563) from public domains were also developed and used for comparison. The mutant plasmid was transformed, and lentiviral production was performed for each CAR mutant. To facilitate the screening, each mutant was displayed on Jurkat cells (T-ALL cell line), and the expression level of the CAR was uniquified by sorting the GFP+ cells from each mutant by flow cytometry and CD3ζ by immunoblot (Additional file 2: Figure S2A and B). The mutant performance was assessed and compared with the parent 74bbz CAR using 4 parameters: (1) functional binding affinity to a chimeric CD74 extracellular domain (ECD)-Fc fusion protein (Additional file 2: Figure S2C); (2) CD69 activation marker expression upon engaging CD74+ target cells; (3) repeated antigen proliferation assay; and (4) in vitro cytotoxicity assay against CD74+ target MCL cell line Mino cells.

### Cell culture and isolation

The MCL cell lines JeKo-1, Mino, Sp53, UPN-1, Granta-519, and Z-138 were obtained from ATCC (Manassas, VA) and cultured under the manufacturer’s instruction. All the cell lines used were routinely tested for mycoplasma with MycoAlert (Lonza, MA) and passaged for no more than 2 months. All the cell lines were regularly STR authenticated. Human peripheral blood mononuclear cells (PBMCs) were isolated from healthy blood donors by Ficoll-Paque Plus (GE Healthcare Life Science, PA) gradient density under an Ohio State University Institutional Review Board approved protocol. Human T cells from peripheral blood were isolated using CD4 and CD8 microbeads in a ratio of 1:1 following the manufacturer’s instructions (Miltenyi Biotech, CA). To determine if activation of T cell and B cells induces CD74 upregulation, T cells were treated overnight with CD3 and CD28 soluble antibodies (10 ng/mL, BioLegend, CA) and 250 U/mL IL-2. B cells were isolated from PBMCs using Easysep human B cell isolation kit (StemCell Technologies, MA) and then treated with LPS (10 ng/mL) and anti-IgM antibody (10 µg/mL). The activation status of the cells was confirmed by flow cytometry. After being isolated from peripheral blood, primary MCL patient cells were cryopreserved and later thawed for use. These cells were cultured in RPMI 1640 with 10% FBS and 5% CO_2_ and used immediately after thawing.

### Antibodies and flow cytometry

Antibodies used in this study included anti-CD3 (clone SK7 and 145-2C11), CD56 (clone N901), CD14 (clone MφP9), human CD45 (clone 2D1), mouse CD45 (clone 30-F11), human LIN (CD3, CD19, CD20, CD56), CD4 (clone RPA-T4), CD69 (clone FN50), CD25 (clone BC96), CD33 (clone WM53), CD11b (QA20A58), and HLA-DR (clone L243). All antibodies are from BioLegend, CA, except CD74 (clone MB741) was from BD Biosciences, CA. Cells were washed once with PBS, blocked with Trustain human Fc blocker (BioLegend), stained with antibodies for 20 min at room temperature and analyzed with a LSRII flow cytometer (BD Biosciences, CA, USA). For parent or mutant CAR-T detection on primary T cells, a truncated EGFR (tEGFR) tag was used in the CAR construct and detected by anti-EGFR antibody (AY13, BioLegend).

For CD74 antigen density determination, molecules of soluble fluorochrome (MESF) FITC-5 premix beads (Bangs Laboratory Inc, IN) were used. These sets of calibrated beads contain a specific number of fluorophore molecules bound per bead. They are used to standardize and convert the mean fluorescence intensity (MFI) in flow cytometry into a count of fluorophores. These allow for the calculation of the number of antigens per cell when using antibodies under saturating conditions, considering the Fluorophore to Protein Ratio (F:P) of each antibody. A calibration curve correlating instrument detection channel values and standardized fluorescence intensity units on constructed with R^2^ of 0.9995. With the F:P ratio of 1 for the CD74 FITC antibody we used, the correlation equation was used to calculate the antigen density of CD74 on MCL cells from the MFI obtained on the same day with settings according to the manufacturer’s instruction.

A flow cytometry based functional binding affinity assay protocol was modified to measure the CD74 scFV on Jurkat cells to CD74 extracellular domain (ECD)-Fc [27, 28]. Briefly, 1 × 10^6^ Jurkat cells (viability > 90%) carrying parent or mutant scFV were washed twice with cold PBS and blocked with Fc blocker (Trustain human Fc blocker, BioLegend) for 10 min at room temperature. The cells were stained with 5 µg/mL CD74-ECD (Sino Biological US Inc., PA) in excess for 45 min on ice, then washed twice with PBS before staining with anti-Fc flow cytometric grade antibody and SYTOX Blue dead cell stain (Invitrogen) for 15 min at room temperature. The cells were washed twice with cold PBS and analyzed immediately by flow cytometry. To confirm the activation of Jurkat cells or primary T cells, the expressions of CD69 and CD25 were determined by flow cytometry.

### Repeated antigen stimulation assay

The stimulation and proliferation methods were performed as previously reported [29]. Jurkat cell or primary T cell clones were co-cultured with irradiated Mino cells (stimulator) at effector-to-target ratio (E:T ratio) of 1:2 at a total cell density of 3 × 10^5^/mL with RPMI 1640 medium containing reduced FBS at 1%. The culture was refreshed with medium every three days and restimulated with freshly irradiated Mino cells for a total of three times before the absolute cell counts were determined by trypan blue exclusion assay. Parent CD74bbz CAR and untransduced-Jurkat or T cells with or without stimulators were used as negative control.

### Cytotoxicity assay

The cytotoxicity of the CAR-T cells was performed with ToxiLight™ non-destructive cytotoxicity bioassay kit (Lonza) and as manufacturer’s instruction described [29]. Briefly, the MCL cell lines or primary MCL patient samples (lymphoma % ranged from 73.7% to 98.7%) were co-cultured with 74bbz CAR-T cells or untransduced T cell control (UTT) cells at E:T ratio of 5:1 for 24 h. At 24 h, the wells for maximum lysis were added with 100% Lysis Buffer (Lonza) for 10 min at room temperature. The volume in other wells were adjusted with the provided Tris AC buffer. The cell supernatant from each well was harvested and reacted with the provided substrate for 5 min before the plates were read for bioluminescence with a Synergy HT microplate reader (Biotek, VT).

### Enzyme-linked immunosorbent assay (ELISA)

Measurement of human IFN-γ in the culture supernatant was performed with ELISA MAX™ Deluxe Set human IFN-γ kit (BioLegend) according to manufacturer’s instruction. Cell-free supernatant was harvested from cell culture 24 h after coculture with effectors (E:T ratio 1:2). The plate was then washed, incubated with tetramethylbenzidine substrate (Agilent Technologies, CA), and read at 450 nm using a Synergy HT microplate reader (Biotek, VT).

### In vivo experiments

All animal studies were approved by The Ohio State University Institutional Animal Care and Use Committee. 4–6-weeks old female NSG mice were used to establish a subcutaneous (s.c.) MCL model using CD74+ Mino cells. On day 0, 1 × 10^6^ Mino cells were mixed with Matrigel matrix (Corning, AZ) at 1:1 v/v and subcutaneously injected in the right flank of the mice. After 3 days, 5 × 10^6^ 74bbz CAR-T cells or UTT control cells were injected intratumorally. The mice were sacrificed when predetermined removal criteria were met (body condition score < 2, hind limb paralysis, and 20% body weight loss). To determine the activity of 74bbz CAR-T cells on human immune cell subsets in vivo, we established a humanized mouse model using CD34+ stem cells purified from human umbilical cord blood as previously described [30]. Briefly, 4-week-old female NSG mice were irradiated at 125 cGy (RS-2000, Rad Source Technologies, GA) and then intravenously injected with 5 × 10^5^ human CD34+ cells, isolated by human CD34 microbead ultra pure kit (Miltenyi Biotech), per mouse. The purities of the human CD34+ cells were > 85% (n = 3). The mice were intraperitoneally injected with stem cell factor (SCF), granulocyte macrophage colony-stimulating factor (GM-CSF), and IL-3 at 4 ng/mL every other day for 3 weeks to support the expansion of the myeloid compartment. After 10-week post-transplant of the human CD34+ cells, human chimerism in circulating cells was determined by facial/submandibular venous blood sampling and flow cytometry. The baseline absolute numbers of human immune cell subsets were collected, and mice were randomized into groups of 5–7 to receive either 5 × 10^5^ UTT or 5 × 10^5^ 42105-74bbz CAR-T cells. The absolute number of human B cells (human CD45+ CD33− CD19+), monocytes (human CD45+LIN−CD11b+CD33+CD14+cells), granulocytic myeloid-derived suppressor cells (G-MDSC, human CD45+LIN−CD11b+CD33+CD14−HLA-DR−), monocytic myeloid-derived suppressor cells (M-MDSC, human LIN−CD45+CD11b+CD33+CD14+HLA-DR−) and NK cells (human CD45+CD33−CD3−CD56+) were measured on Day 3, 11, 18, 23 post UTT/CAR-T injections. The UTT and CAR-T cells were traced and identified as human CD45+CD3+ and CD45+CD3+EGFR+, respectively.

### Statistical analysis

For independent data, two-sample t-test or ANOVA were used for two group comparisons or for multiple group comparisons, respectively. For paired or correlated data, such as cells from the same subjects treated with different treatment conditions, paired t-tests or linear mixed effects models were used to adjust the correlation among observations from the same object. To test the dependence of antigen expression on cytotoxicity, linear regression model was used. For survival studies, log rank test was used for comparison and survival curves were displayed with Kaplan–Meier survival curves. *P*-values were adjusted for multiple comparisons by Holm’s procedure when needed. A *p-*value less than 0.05 for two group comparisons or after adjustment for multiple comparisons was considered statistically significant.

## Results

### scFV optimization of CD74bbz CAR identifies a clone of CAR-T cell with maximal target induced proliferation and cytotoxicity.

**Fig. 1:**
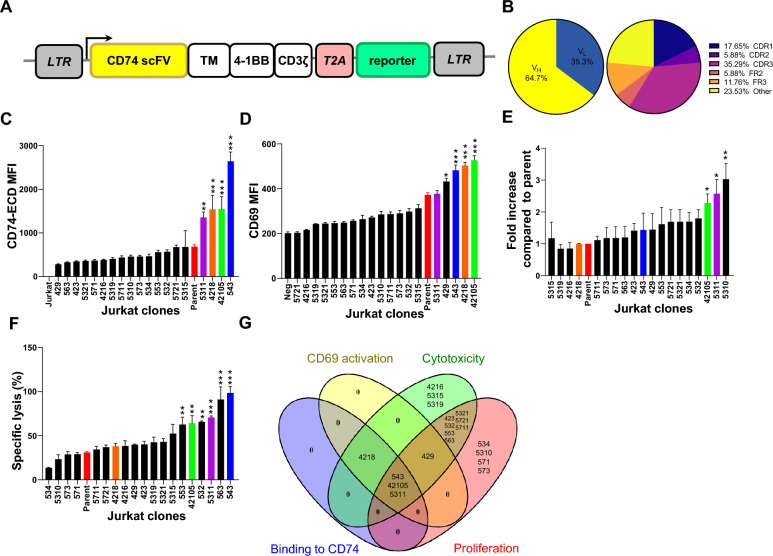
scFV sequence optimization yields an anti-CD74 CAR (74bbz)-T cell with superior functions. **A** Design of CD74 CAR lentiviral vector. CD74 scFV: anti-CD74 scFV ended with CD8α hinge region; TM: CD8α transmembrane domain; 4-1BB: signaling domain of 4-1BB molecule; CD3ζ: CD3 zeta chain; T2A: self-cleaving peptide from thosea asigna virus 2A; reporter: GFP or tEGFR reporter gene. **B** Distribution of mutations on the V_H_/V_L_ chain (right) and framework (FR)/complementarity-determining region (CDR) of the scFV on the 74bbz CAR. **C** Binding affinity of 74bbz CAR mutants displayed on Jurkat cell to CD74 ECD-Fc. The Jurkat CAR mutant cells were stained with CD74-ECD-Fc followed by anti-Fc-APC for flow cytometric analysis. The red bar is the parent 74bbz. All the numbers on the x-axis are the CAR clone numbers. **D** Early T cell activation marker CD69 expression on Jurkat CAR mutant cells. The Jurkat CAR mutant cells were incubated with Mino cells at an effector-to-target (ET) ratio of 1:2 for 6 h before the Jurkat CAR mutant cells were stained for CD69. **E** Repeated antigen stimulation assay for Jurkat CAR mutant cells. The Jurkat CAR mutant cells were stimulated weekly with irradiated Mino cells for three weeks and cultured under suboptimal culture condition with reduced FBS at 1%. The cell proliferation on Jurkat CAR mutant cells were assayed and the percentage of increased cell numbers were normalized as fold increase compared to Jurkat cells with parent 74bbz cells. **F** Specific lysis of the Mino cells by Jurkat CAR mutant cells. Jurkat CAR mutant cells were cocultured with Mino cells at E:T ratio of 5:1 for 24 h before the specific lysis were analyzed. **G** A four-way Venn diagram identified 2 clones of 74bbz CAR with the superiority over four aspects of CAR-T cell: CD69 activation (yellow), cytotoxicity (green), proliferation (red), and binding to CD74 (blue)

**Fig. 2:**
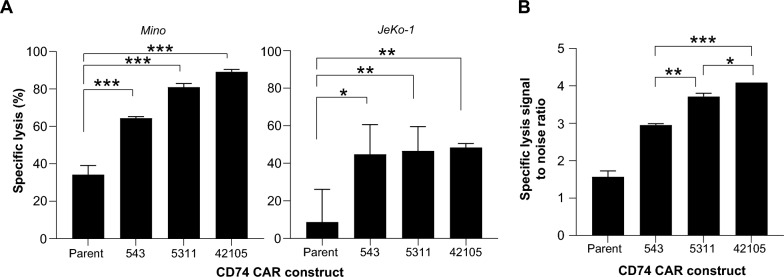
42105-74bbz CAR-T cells induced the highest specific lysis among mutants against Mino and JeKo-1 cells. **A** Three 74bbz CAR mutants were expressed on primary CD4/8 T cells (n = 3) and used as effector cells in the co-culture of Mino (left) and JeKo-1 (right). Mutants 543, 5311 and 42105 were compared to the parent 74bbz CAR-T cells. **B** 42105-74bbz CAR-T cells had the highest specific lysis signal-to-noise as determined by the specific lysis on CD74+ Mino cells over the CD74- SUDHL-1 cells. The ratios from all three mutants were compared to that of the parent. Data are mean ± S.E.M. from three independent experiments. * *p* < 0.05; ** *p* < 0.01; ***p < 0.001

We designed a second-generation CAR construct with 4-1BB and CD3ζ chain signaling domains by joining a scFV specifically targeting human CD74 with the CD8 hinge/transmembrane domain and CD3ζ (Fig. 1A). Recent reports suggest the role of framework and CDR regions in the scFV domains on the tonic signaling and persistence of CAR-T cells. We performed an optimization of the CDR region of scFV and aimed to select the best functional CD74bbz CAR-T cells utilizing an in silico approach and functional assays. We created an anti-CD74 scFV-CD74 antigen docking model and refined it with alanine scanning (Additional file 1: Figure S1A and B). For V_H_, 4 positions in CDR1, 10 in CDR2, and 8 in CDR3 were found to be important for the CD74 binding interaction. For V_L_, 5 positions in CDR1, 2 in CDR2, and 6 in CDR3 were those important residues for interacting with CD74 antigen. We then mutated the 22 amino acid residues in the V_H_ and 13 in the V_L_ using mCSM-AB2, a computational application to rationally assess the impact of single-point mutations on the binding affinity between antibodies and antigens with high accuracy [30]. A total of 741 mutants were successfully tested and the potential CD74 binding energy difference upon mutation between the wild-type and the mutants were ranked in ∆∆G (kcal/mol). A total of 17 mutants were chosen based on the positive energy enhancement compared to the wild type. 64.7% of the 17 mutants were on V_H_ while 35.3% were on V_L_ (Fig. 1B, left). In addition, 35.29% of the 17 mutans were on CDR3 regions, 17.65% in CDR1, and 5.88% in CDR2 (Fig. 1B, right). We engineered a T-cell acute lymphoid leukemia cell line (Jurkat) to express the selected mutant constructs. The expression of CAR on each mutant clone was normalized by sorting each Jurkat clone at the same intensity of the GFP and confirmed by immunoblotting (Additional file 2: Figure S2A and B). We tested the binding affinity of each mutant CAR to a chimeric CD74 ECD-Fc by flow cytometry [28] and 4 clones (5311, 4218, 42105, and 543) were found to have increased binding affinity to the CD74 antigen compared to the parent (Fig. 1C). We then evaluated the expression of the T cell activation marker CD69 and identified 4 clones (429, 4218, 543 and 42105) with significantly increased CD69 expression 8 h after co-incubation with the MCL cell line Mino cells at an E:T ratio of 1:2 (Fig. 1D). Following the repeated antigen stimulation assay, clones 42105, 5311, and 5310 displayed a significantly higher fold increase in cell number compared to the parent CAR (Fig. 1E). Similarly, clones 543, 563, 5311, 532, 42105 and 553 induced significantly higher specific lysis compared to parent CAR-T cells (Fig. 1F). By including all clones with augmented function compared to the parent CAR-T cells (with or without statistically significant increase) in a Venn analysis, clones 42105, 5311, and 543 were identified as the three lead candidates for further screening as they met all the 4 proposed criteria including CD69 activation, CD74 binding affinity, target induced proliferation, and cytotoxicity (Fig. 1G). Using primary T cells (n = 3) as effectors and MCL cell lines Mino (CD74 high expressor) and JeKo-1 (CD74 low expressor) as target cells, all 3 mutants induced significantly higher specific lysis compared to the parent 74bbz CAR-T cells (Mino, Fig. 2A, left; JeKo-1, Fig. 2A, right). To determine if the mutant clones were specific to CD74, we measured the specific lysis on CD74 positive Mino cells (on-target signal) over a CD74 negative T cell lymphoma cell line SUDHL-1 (off-target noise) and compared the 3 mutant clones to the parent. 42105 showed significantly higher signal to noise ratio than 5311 and 543 (Fig. 2B), prompting us to choose clone 42105 (42105-74bbz CAR) for our studies.

### CD74+ MCL cells are sensitive to 42105-74bbz CAR-T cells

**Fig. 3:**
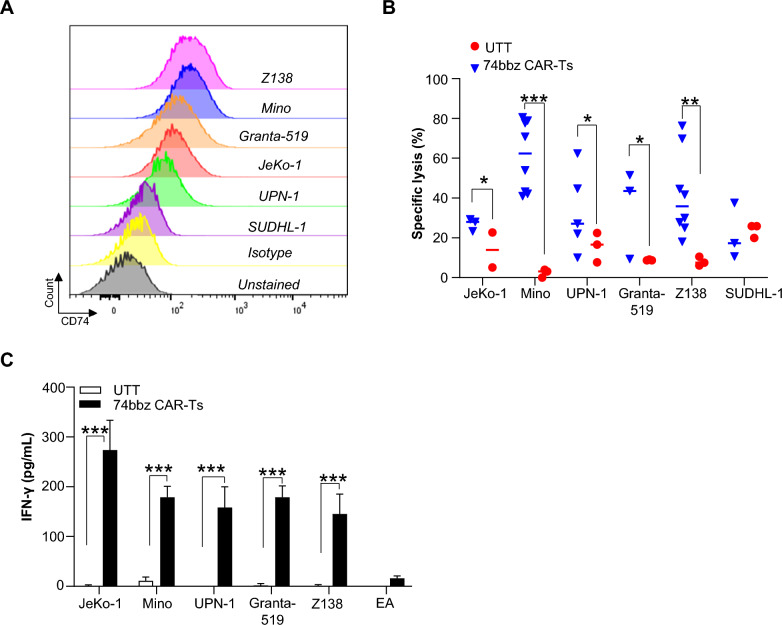
Cytotoxicity and IFN-γ production of optimized 42105-74bbz CAR-T cells against MCL cells. **A** Expression of CD74 on MCL cell lines. The data presented are one representative of three independent experiments. Unstained and isotype controls were used as a staining negative control. **B** Cytotoxicity assay of optimized 42105-74bbz CAR-T cells against 6 different MCL cell lines. Untransduced T cells (UTT, red circle) or optimized 42105-74bbz CAR-T cells (blue, triangles) were co-cocultured with target cells at E:T ratio of 5:1 for 24 h before the specific lysis was determined. CD74− T cell lymphoma cell line SUDHL-1 was used as a negative control. Each dot represents one experimental result from each healthy donor CAR-T cells. **C** IFN-γ production from 42105-74bbz CAR-T cells after co-cultured with indicated cell for 24 h. Effector alone (EA) was used as the negative control. Results were presented as mean ± SEM from three independent experiments. * *p* < 0.05; ** *p* < 0.01; ***p < 0.001

**Fig. 4:**
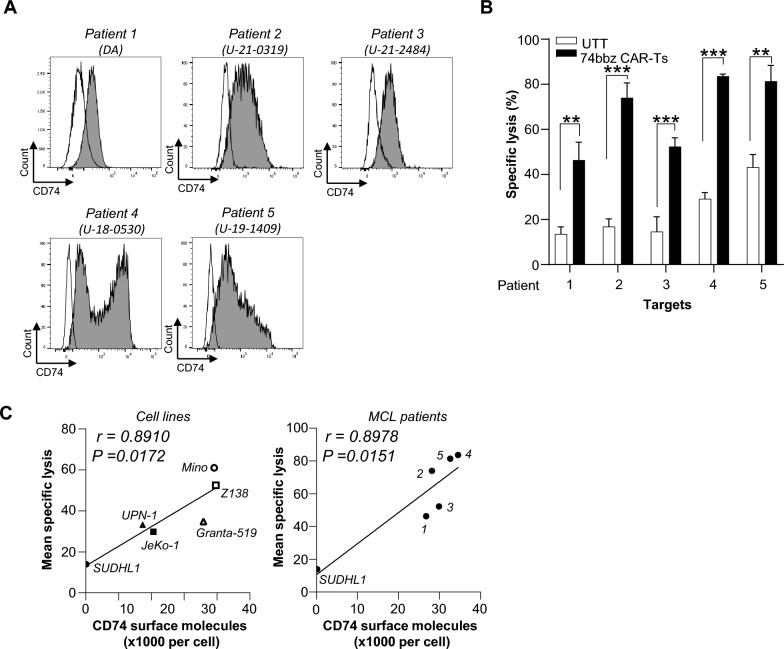
Activity of optimized 42105-74bbz CAR-T cells against primary MCL patient lymphoma cells. **A** Expression of CD74 (gray) on MCL patients’ lymphoma cells compared to isotype control (white). The data presented are one representative of three independent experiments. **B** Susceptibility of five MCL patient lymphoma cells to the 42105-74bbz CAR-T cells. MCL patient cells were used as target cells in the co-culture of optimized 74bbz CAR-T cells at E:T ratio of 5:1 for 24 h. Results were presented as mean ± SEM from three independent experiments. ** *p* < 0.01; ***p < 0.001. **C** Antigenic dependence of CD74 on the specific lysis of 42105-74bbz CAR-T cells. The CD74 surface antigen density estimated by MESF and specific lysis values of JeKo-1, Mino, UPN-1, Granta-519, Z138 (left), and Patient 1–5 (right) including negative control SUDHL-1 were used to perform the correlation analysis for the correlation coefficient r and the *p*-value

To assess the sensitivity of MCL cells to 42105-74bbz CAR-T cells, we performed cytotoxicity assays in 6 CD74 positive MCL cell lines (JeKo-1, Mino, UPN-1, Granta-519, and Z138) and a CD74 negative T cell lymphoma cell line SUDHL1 as negative control (Fig. 3A). 42105-74bbz CAR-T cells were generated using purified T cells from healthy donors with an average transduction efficiency at 3 days of 73.03 ± 11.4%. We found that all the MCL cell lines were susceptible to the 42105-74bbz CAR-T cells with significant specific lyses compared to UTT control. A median of 28.1%, 62.4%, 43.3%, 43.6%, and 35.8% of JeKo-1, Mino, UPN-1, Granta-519, and Z138, respectively, were lysed at 24 h with an E:T ratio of 5:1 (Fig. 3B). Engaging 42105-74bbz CAR-T cells with the indicated target MCL cell lines induced T cell activation with a significant increase in IFN-γ production after 24 h of co-culture compared to the UTT and effector T cell negative controls (Fig. 3C). Five primary CD74+/CD19+/CD5+ MCL patient samples were used for further validation (clinical characteristics of these 5 MCL patients are summarized in Table 1) (Fig. 4A). Co-culture of 42105-74bbz CAR-T cells with the primary MCL patient cells (n = 5) at E:T ratio of 5:1 for 24 h resulted in significant lysis compared to UTT control in all the samples tested including one which relapsed after commercial CD19 CAR-T cell therapy (Fig. 4B). Further analyses on the MCL cell lines (Fig. 4C, left) and primary MCL patient samples (Fig. 4C, right), including CD74 negative control SUDHL-1, showed a positive correlation between target antigen expression and cytotoxicity (Correlation coefficient r of 0.8910 and 0.8978, respectively). These results indicate that MCL are sensitive to the effector functions of 42105-74bbz CAR-T cells.

**Table 1:** Clinical characteristics of MCL patients

Patient ID	Diagnosis	Treatment	Immunophenotype
1 (DA)	MCL	Treatment naive	CD5+CD19+ (86.1%+)
2 (U-21-0319)	MCL	Treatment naive	CD5+CD19+ (85.5%+)
3 (U-21-2484)	MCL	R/R status post chemotherapy including ASCT, targeted therapies including ibrutinib and venetoclax, and progressed after commercial CD19 CAR-Ts	CD5+CD19+ (95.2%+)
4 (U-18-0530)	MCL	Treatment naive	CD5+CD19+ (85.4%+)
5 (U-19-1409)	MCL	R/R status post chemotherapy and ibrutinib	CD5−CD19+ (73.7%+)

### 42105-74bbz CAR-T cells are minimally cytotoxic against most normal immune cell subsets

**Fig. 5:**
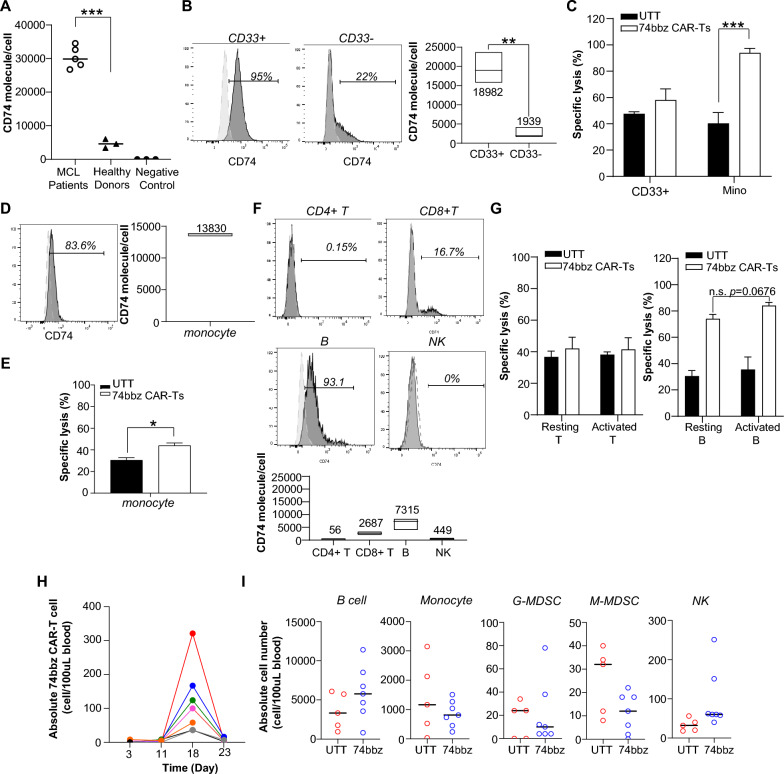
CD74 expression remains on CD33+ myeloid subset of normal PBMCs. **A** CD74 antigen density on MCL cell (Table 1) was significantly higher than PBMCs from healthy blood donors, while no statistically significance between PBMCs and negative control SUDHL-1 was found. **B** Histogram plots of CD74 expression on CD33+ and CD33− PBMCs (shaded gray) compared to the isotype staining control (dotted line). CD74 densities on CD33+ and CD33− PBMCs were summarized from 3 independent donors (right). **C** Specific lysis of normal CD33+ cells when used as target cells for 42105-74bbz CAR-T cells. Mino cells were used as a positive control target. **D** CD74 expression on CD33+CD14+ monocyte (shaded gray) compared to the isotype staining control (dotted line). A summary of CD74 density on monocytes from 3 independent donors was plotted (right). **E** Susceptibility of monocytes to 42105-74bbz CAR-T cells (white) and UTT (black). **F** A subset of CD33−CD3+CD4+ T cells, CD33−CD3+CD8+ T cells, CD33−CD3−HLA-DR+CD19+ B cells and CD33−CD3−CD56+ NK cells expressed CD74. Data are from one representative of three independent experiments. CD74 densities on B cells, CD4+ T cells, CD8+ T cells and NK cells were all below 10,000 molecules per cell. Numbers indicate the median molecules per cell. Data are from three independent donors (bottom). **G** Susceptibility of resting and activated T/B cells to 42105-74bbz CAR-T cells (white) and UTT cells (black). Results are mean ± S.E.M. from three independent experiments. * *p* < 0.05; ** *p* < 0.01; ***p < 0.001. **H** 42105-74bbz CAR-T cells did not deplete normal immune cells. Autologous 42105-74bbz CAR-T cell number peaked on Day 18 post CAR-T cell injection in the humanized NSG mice. The absolute numbers of 74bbz CAR-T cell in these mice are plotted over time in different colors. **I** And there were no significant changes on the absolute cell numbers of circulating B cells, monocytes, G-MDSC, M-MDSC and NK cells measured on the same day. All human cells were identified by human CD45+. B: CD33−CD19+; Monocyte: LIN−CD45+CD11b+CD33+CD14+; G-MDSC: LIN−CD11b+CD33+CD14−HLA-DR−; M-MDSC: LIN−CD11b+CD33+CD14+HLA-DR−; NK: CD33−CD3−CD56+. Mice received either UTT cells (red, n = 5) and 42105-74bbz (blue, n = 7). Bars show the median cell number

To differentiate the activity of 42105-74bbz CAR-T cells on MCL and non-malignant immune cells, we measured the CD74 density per cell by MESF estimation in PBMCs from healthy blood donors (n = 3) and primary MCL patient samples (n = 5). PBMCs from healthy blood donors had less than 10,000 molecules per cell, values which were significantly lower than MCL cells, which ranged from 26,752 to 34,561 molecules per cell (Fig. 5A, p = 0.0019). There is no statistical difference between normal cells and negative control SUDHL1 cells (*p* = 0.656). Within the PBMCs, CD33+ myeloid cells had higher percent positive and CD74 molecules per cell than the CD33− subset (95% vs 22% CD74+, Fig. 5B). Importantly, 42105-74bbz CAR-T cells were minimally cytotoxic against CD33+ cells (Fig. 5C, p = 0.456). Within CD33+ population, majority of monocytes (83.6%) were CD74 + and had a median of 13,830 CD74 molecules per cell (Fig. 5D). Using purified monocytes as the target cell in a coculture experiment with 42105-74bbz CAR-T cells using UTT cells as control, a modest but significant increase in specific lysis was observed, indicating that these cells are susceptible to 42105-74bbz CAR-T cell cytolysis (Fig. 5E). Among the lymphoid cells, most of the normal CD33−HLA-DR+CD19+ B cells (93.1%) were CD74+ with a median density of 7315 molecules per cell while only a subset of CD33−CD3+CD8+ T cells (16.7% CD74+, median density 2687 molecules per cell), CD33−CD3+CD4+ T cells (0.15% CD74+, median density 56 molecules per cell) and CD33−CD3−CD56+ NK cells (0% CD74+ based on compared with isotype control, median density 449 molecules per cell) expressed CD74 (Fig. 5F). Of note, activation of T cells (CD3/CD28 soluble antibodies and IL-2) and B cells (LPS, 10 ng/mL, and anti-IgM, 10 µg/mL) did not affect CD74 expression or CD74-sepcific lysis (Additional file 3: Figure S3 and Fig. 5G). To characterize the activity of 42105-74bbz CAR-T cells on normal immune cell in vivo, a CD34+ hematopoietic stem cell humanized mouse model was developed and utilized [30]. Human CD74−CD34+ stem cells were isolated from umbilical cord blood (Additional file 4: Figure S4) and engrafted in NSG mice. The mice were intraperitoneally injected with SCF, GM-CSF and IL-3 to promote the myeloid differentiation as previously reported [30]. After 10 weeks, human CD45+ cell reconstitution as determined in peripheral blood, mice were randomized to receive either 5 × 10^6^ autologous 42105-74bbz CAR-T cells generated from the same cord blood donor or UTT cells intravenously. As shown in Fig. 5H, 74bbz CAR-T cells, which peaked at Day 18 post CAR-T injections, did not have a significant effect on the absolute numbers of circulating human B cells, monocytes, G-MDSC, M-MDSCs and NK cells (Fig. 5I and Additional file 5: Figure S5). Collectively, these in vitro and in vivo results show that while 74bbz CAR-T cells are minimally cytotoxic against most of normal immune cell subsets, their cytotoxicity appears to correlate with target antigen density.

### 42105-74bbz CAR-T cells show strong antitumor activity in a MCL xenograft tumor model

**Fig. 6:**
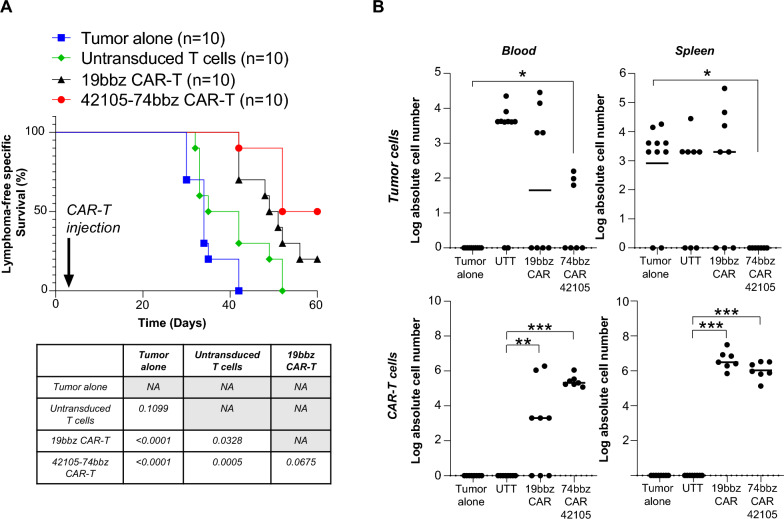
42105-74bbz CAR-T cells are not inferior to the 19bbz CAR-T cells in prolonging the survival in a preclinical MCL NSG mouse model. **A** Mice were engrafted with Mino cells subcutaneously and randomized into groups of 10 for tumor alone (blue), tumor + UTT cells (green), tumor + 19bbz CAR-T cells (black) and tumor + 42105-74bbz CAR-T cells (red). Table (bottom) was a summary of *p*-values in survival comparisons among all groups. **B** Mice received 42105-74bbz CAR-T cells had the least median log absolute numbers of tumor cells in blood and spleen harvested when mice reached ERC. While the median log absolute number of circulating CAR-T cells in 74bbz CAR-T cells treated mice was higher than that of 19bbz CAR-T cells treated mice, similar numbers of CAR-Ts in both 19bbz and 74bbz CAR-T cells treated mice were found in spleen

To determine if 42105-74bbz CAR-T treatment was non-inferior to the standard CD19 CAR-T therapy, we adopted a subcutaneous MCL xenograft model using NSG mice engrafted with Mino cells. The animals in groups of 10 were randomized to receive tumor alone, tumor + 5 × 10^6^ UTT cells, tumor + 5 × 10^6^ 19bbz CAR-T cells, and tumor + 5 × 10^6^ 42105-74bbz CAR-T cells intratumorally 3 days after 1 × 10^6^ Mino cells were engrafted and when palpable tumors were detected. As shown in Fig. 6A, treatment with both 19bbz CAR-T cells and 42105-74bbz CAR-T cells significantly prolonged the mice survival compared to the tumor alone (*p* < 0.0001, n = 10) and UTT groups (*p* < 0.0001 compared to untreated group, n = 10) without evidence of significant body weight loss (Additional file 6: Figure S6). In addition, mice treated with 42105-74bbz CAR-T cells had a median survival of 56 days (n = 10, 50% survived at end-point) compared to 50 days for those treated with 19bbz CAR-T cells (n = 10, 20% survived at end-point); however the survival advantage did not reach statistical significance (*p* = 0.0675) despite the fact that mice treated with 42105-74bbz CAR-T cells had the lowest number of MCL cells in both peripheral blood and spleen (Fig. 6B). Circulating tumor cells were not detected in tumor alone group, except at the injection site at early removal criteria (ERC). The circulating and spleen infiltrating CAR-T cells in the 42105-74bbz CAR-T cell treated mice remained high and comparable between the 2 CAR-T cell treated groups (Fig. 6B, p > 0.05). Collectively, our data provide evidence that 42105-74bbz CAR-T is highly active in preclinical models of MCL and is a novel cell therapy product warranting further development.

## Discussion

Currently, the cell therapy-based approach for MCL patients primarily focuses on CD19 as a target. In 2020, the US Food and Drug Administration (FDA) approved brexucabtagene autoleucel (Tecartus) which is a CD19 targeting CAR-T cell therapy for the treatment of MCL assessed in the ZUMA-2 clinical trial [11]. After a median follow-up of ~ 3 years, the PFS was 25.8 months, suggesting that a significant portion of these patients will need additional treatment. In this manuscript, we present the development, characterization, and optimization of a first-in-class CAR-T cell therapy targeting CD74, an invariant chain of MHC class II HLA-DR, which is overexpressed on MCL cells. 42105-74bbz CAR-T cells showed significant cytotoxicity against MCL cells in vitro including a primary MCL patient sample that relapsed after commercial CD19 CAR-T cell therapy. While CD74 is also expressed at a lower level on normal immune cells, we provide convincing evidence of minimal cytotoxicity of 42105-74bbz CAR-T cells on normal immune cells in vitro and in vivo.

The current treatment of MCL involves a multi-modal approach that includes chemotherapy, stem cell transplant, and targeted therapies [6]. NCCN guidelines recommend an intensive chemotherapy regimen followed by autologous stem cell transplantation and maintenance rituximab for patients who are physically fit and under 65. For the majority of MCL patients, who are over 65, less aggressive treatment strategies are adopted [31]. Small molecule inhibitors of BTK including ibrutinib, acalabrutinib, zanubrutinib, and pirtobrutinib, are FDA-approved for the treatment of relapsed/refractory MCL and provide significant clinical benefits for a period of time. Unfortunately, the vast majority of MCL patients treated with targeted therapies eventually progress and develop a more aggressive disease, resulting in low response rates to other treatment approaches and an overall short survival. Anti-CD19 KTE-X19 CAR-T cells (Tecartus) represent a promising cell therapy option for relapsed and refractory MCL. The overall response rate and complete remission rates following treatment of MCL patients with Tecartus was high at 91% and 68%, respectively, however, the median PFS and OS with approximately 3 years of follow up were 25.8 and 46.6 months, respectively, thus suggesting that a significant portion of these patients will need additional treatment.

As reported by Wang et al. in their initial study, the patients who received KTE-X19 CAR-T cells experienced predictable CAR-T therapy adverse events, such as cytopenias (grade 3 or higher in 94% of the patients), cytokine release syndrome (91% of patients, 15% of which were grade 3–4), and neurotoxicity (63% of patients, 31% of which were grade 3–4), which might be mediated by the expression of CD19 on brain pericytes [32]. Interestingly, analysis of the same data set revealed that *CD74* is the least differentially expressed gene on brain pericytes relative to B cells. We confirmed absence of CD74 surface expression on primary brain perocytes using flow cytometry (data not shown). In our subcutaneous MCL model, the mice treated with 42105-74bbz CAR-T cells had significantly lower number of MCL cells in both the peripheral blood and spleen as shown in Fig. 6B, although the survival advantage was not statistically significant between 42105-74bbz treated and 19bbz treated mice (*p* = 0.0675). This suggests that targeting CD74 with CAR-T therapy is comparable in efficacy to targeting CD19.

Our group has contributed to the characterization of CD74 as a therapeutic target and pioneered the preclinical development of milatuzumab, a naked fully humanized monoclonal antibody targeting CD74, in MCL [19–21]. While single agent milatuzumab has shown significant activity in preclinical models of B-cell malignancies, milatuzmab has not provided meaningful clinical benefits to patients with CLL, MM, and B-cell NHL, primarily due to its very short half life secondary to rapid internalization of the antibody-antigen complex and antigen sink [18, 23]. Given the potenial of CD74 as a novel therapeutic target, we designed a second-generation CAR construct with 4-1BB and CD3ζ chain signaling domains by joining a scFV specifically targeting human CD74 with the CD8 hinge/transmembrane domain and CD3ζ and transduced the construct in CD74 negative CD4/CD8 (1:1) minimizing the risk of CAR-Ts fratricide effect. In addition, our CAR-T cell construct contains a humanized scFV minimizing its immunogenicity and the risk for anti-CAR immunity [32].

In our study, we adopted a strategy for the optimization of our 74bbz CAR-T cells, utilizing a variety of assays, including a flow cytometry-based antigen binding assay, T cell activation assay, repeated antigen stimulation assay [29], and cytotoxicity assay. Through in silico modeling of the binding of CD74 antigen to anti-CD74 scFV mutants, we found that mutations on the V_H_ chain and CDR3 had the highest docking scores and produced mutants with higher T cell activation. However, increased binding affinity and T cell activation did not always lead to improved cytotoxicity and cell expansion (Fig. 1). While it remains unclear which of the four factors we tested is most critical to clinical use, our optimization strategies provided a rational methodology for screening CARs for potential clinical applications.

The main limitation of targeting CD74 is its expression on antigen presenting cells such as B cells and monocytes, in addition to a subset of myeloid cells [15, 23]. Interestingly, milatuzumab showed a favorable safety profile in clinical trials with the most common adverse event being infusion reactions. In the phase I–II clinical trial for patients with relapsed/refractory CLL, treatment with milatuzumab did not result in significant cytopenias. Notably, a significant increase in non-malignant lymphocyte counts following each infusion of milatuzumab was observed in patients who obtained a transient response to milatuzumab [18]. In the phase I dose escalation clinical trial with single agent milatuzumab in patients with relapsed/refractory MM, no significant changes were seen from baseline [33]. In the phase I study with milatuzumab monotherapy in patients with relapsed B-cell NHL, the most common treatment-related grade 3–4 hematologic toxicities were neutropenia (9%) and thrombocytopenia (5%), but not lymphopenia [23].

Overall, our data show that CD74 expression is significantly lower in normal immune cell subsets compared to MCL cells. Among the normal immune cell subsets, myeloid and B cells expressed the highest levels of CD74 compared with CD4+ T and CD8+ T cells. Interestingly, our 42105-74bbz CAR-T cells failed to induce significant cytotoxicity in resting and activated normal T cells and CD33+ myeloid cells, while cell killing was observed in resting and activated B cells in vitro. However, we did not see any significant depletion of immune cell subsets in peripheral blood including B cells, monocytes, and NK cells in our humanized mouse model.

More work is needed to characterize the differential cytotoxic effect of 42105-74bbz CAR-T cells on immune cells subsets, however we speculate that these differences could be partially explained by the pro-survival function played by CD74 in normal B cells. Specifically, reports suggest that CD74 is indispensable for the proper B cell development and, together with CD44, promotes B cell proliferation and survival by activation of downstream pathways, such as NF-kB and PI3K/Akt signaling upon MIF binding [12]. We hypothesize that 42105-74bbz CAR-T cells exert a direct inhibitory effect on the pro-survival signal mediated by CD74. CD74 can also be expressed by epithelial cells of the gastrointestinal (GI) tract under inflammatory conditions [15, 34], which is obviously a concern for potential toxicity following treatment with 42105-74bbz CAR-T cells. CD74 is post-translationally glycosylated and, in humans, it exists in 5 isoforms [12]. While no GI toxicities were reported in the milatuzumab clinical trials, more work is needed to clarify this important aspect of CD74 targeting and to understand if the expression of CD74 isoforms is tissue-specific.

An interesting and relatively unexplored aspect of CD74 as a therapeutic target is that its surface expression can be pharmacologically modulated by inhibiting its degradation in the autophagosme/lysosomal compartment [19, 21]. We have previously shown that the enhancement of CD74 expression positively correlated with milatuzumab-mediated MCL cell death [19, 21]. Work by others [27, 35] and our data presented here indicate that the cytotoxicity activity of CAR-T cells correlates positively with target antigen density, thus providing rationale for combination strategies to maximize the therapeutic potential of 42105-74bbz CAR-T cells. From a toxicity standpoint, it is important to note that inhibition of CD74 degradation affects the expression of CD74 on lymphoma cells while leaving its expression on normal immune cells unaffected, probably because lymphoma cells rely more heavily on autophagy for survival as compared to normal immune cells [36]. In conclusion, we provide the preclinical evidences showing anti-CD74 CAR-T cell therapy is an effective treatment approach for MCL patients warranting further development and exploration of combination therapies to augment tumor-specific CD74 expression.

## Conclusions

This is the first study describing the development and optimization of a CD74bbz CAR-T cell product. Our findings provide evidence of significant activity of 42105-74bbz CAR-T cells in preclinical MCL models with minimal toxicity in normal immune cell subsets and pave the way for the clinical development of this product.

### Supplementary Information

**Additional file 1: Figure S1.** In silico modeling of CD74-anti-CD74 scFV interaction. **A** Best generated models of CD74-anti-CD74 scFV interaction shown by the lowest HADDOCK score as a function of RMSD. The blue cluster was picked for further in silico mutagenesis. **B** Visualization of CD74-anti-CD74 scFV interaction. Red: CD74 trimer; Blue: anti-CD74 scFV.

**Additional file 2: Figure S2.** Creation of the 74bbz mutant clones. **A** GFP+ cells of the 74bbz mutants and parent CAR expressing Jurkat cells were sorted at the same intensity by flow cytometry. **B** An immunoblot of CD3ζ to show the expressing of parent, 543, 5311, 42105-74bbz clones. Endogenous CD3ζ was detected at 15 kDa while the chimeric CD3ζ on CAR was detected at 55 kDa. **C** CD74-ECD-Fc fusion protein was stained by Coomassie blue staining.

**Additional file 3: Figure S3.** Expression of CD74 after activation on T cell and B cell. T cells and B cells isolated from PBMCs of 3 healthy blood donors were either untreated (red) or activated (blue) by CD3/CD28 soluble antibodies and IL-2 for T cells, and LPS (10 ng/mL)/ anti-IgM (10 µg/mL) for B cells. One representative of 3 healthy blood donors was shown.

**Additional file 4: Figure S4.** Expression of CD74 on human CD34+ stem cells. Human umbilical cord blood mononuclear cells were stained with anti-CD34 and CD74 antibodies and analyzed by flow cytometry. One representative of 3 healthy cord blood donors was shown.

**Additional file 5: Figure S5.** No significant depletion of circulating immune cells pre/post peak detection of 42105-74bbz CAR-T cells in a humanized mouse model. **A** Absolute cell numbers of B cells, monocytes, G-MDSC, M-MDSC and NK cells in humanized NSG mice on Day 3, 11 and 23 post UTT or 74bbz CAR-T engraftment. All human cells were identified by human CD45+. B: CD33−CD19+; Monocyte: LIN−CD45+CD11b+CD33+CD14+; G-MDSC: LIN−CD11b+CD33+CD14−HLA-DR−; M-MDSC: LIN−CD11b+CD33+CD14+HLA-DR−; NK: CD33−CD3−CD56+. Mice received either UTT cells (red, n = 5) and 42105-74bbz (blue, n = 7). Bars show the median cell number. **B** CD74 expression (blue) of B cells, monocytes, G-MDSC, M-MDSC and NK cells on Day 18 compared to isotype control (red). Data are from one mouse from the 42105-74bbz CAR-T cell treatment group.

**Additional file 6: Figure S6.** No significant change in the body weights of the 74bbz CAR-T cells-treated mice was observed. The body weights of the mice treated UTT, 19bbz CAR-T and 74bbz CAR-T cells (n = 10 per group) were monitored until the mice reached ERC and plotted over time.

## Data Availability

Not applicable.

## Abbreviations

42105-74bbz: Clone 42105 of anti-CD74 CAR with 4-1BB and CD3ζ signaling domains
74bbz: Anti-CD74 CAR with 4-1BB and CD3ζ signaling domains
BTK: Bruton Tyrosine Kinase
CD19bbz: Anti-CD19 CAR with 4-1BB and CD3ζ signaling domains
CDR: Complementarity-determining region
CLL: Chronic lymphocytic leukemia
δδG: Delta delta energy
ECD: Extracellular domain
ERC: Early removal criteria
ELISA: Enzyme-linked immunosorbent assay
ER: Endoplasmic reticulum
ET: Effector-to-target
GM-CSF: Granulocyte macrophage colony-stimulating factor
G-MDSC: Granulocytic myeloid-derived suppressor cells
IFN-γ: Interferon-gamma
IL-3: Interleukin-3
MCL: Mantle cell lymphoma
MESF: Molecules of Equivalent Soluble Fluorochrome
MIF: Macrophage migration inhibitory factor
MM: Multiple myeloma
M-MDSC: Monocytic myeloid-derived suppressor cells
NK cell: Natural killer cell
NSG: NOD-SCIDγc−/− mouse
S.C.: Subcutaneously
SCF: Stem cell factor
scFV: Single chain variable fragments
SPPL2a: Signal Peptide Peptidase-Like 2A
UTT: Untransduced T cell control
VH: Heavy chain
VL: Light chain
