# Supplementary figures and images for: A novel CAR-T cell product targeting CD74 is an effective therapeutic approach in preclinical mantle cell lymphoma models
Source: Exp Hematol Oncol. 2023 Sep 22;12:79. doi: 10.1186/s40164-023-00437-8 (PMC10517521; doi:10.1186/s40164-023-00437-8)

## Slide 1
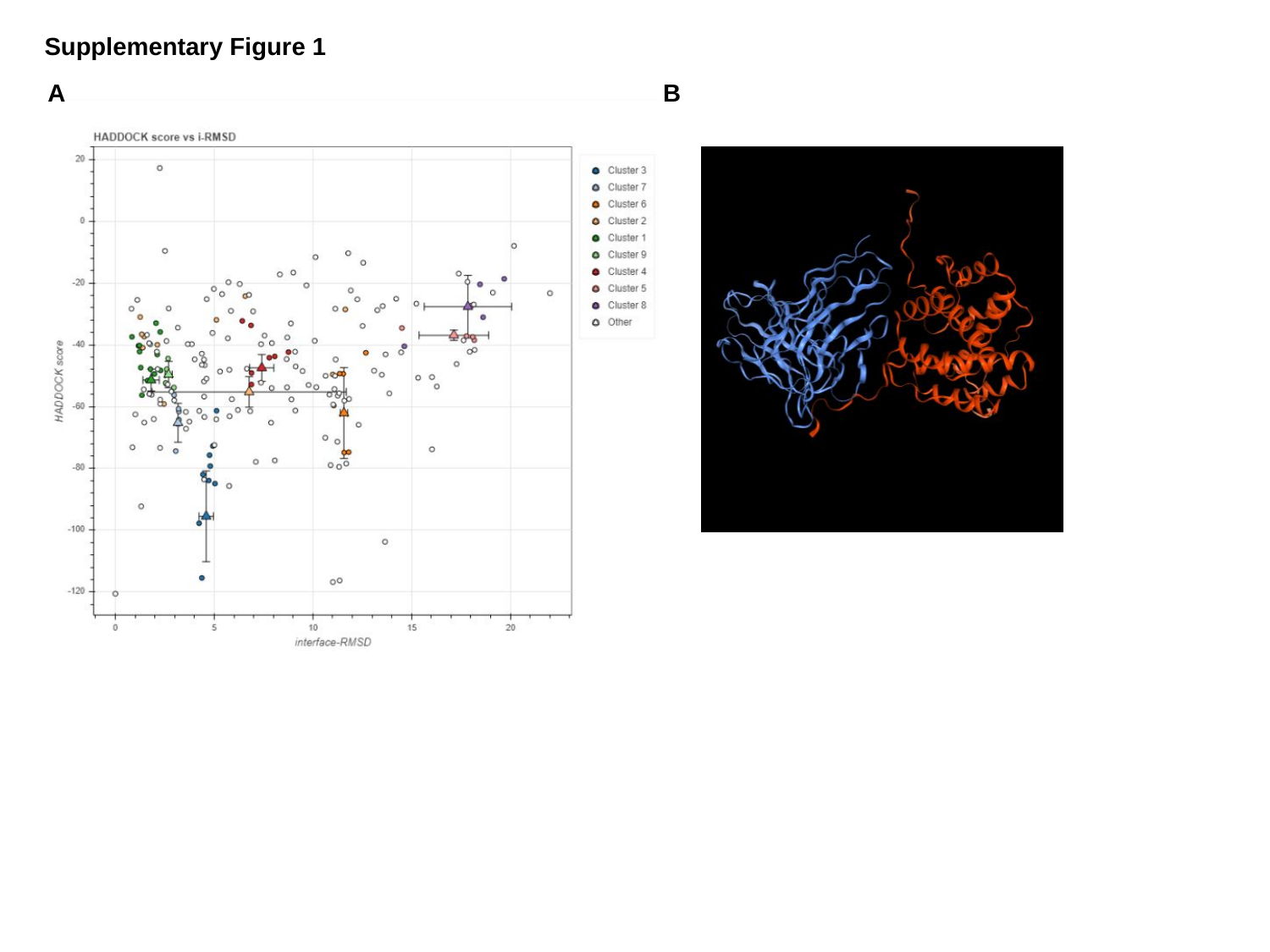

Supplementary Figure 1
A
B

Supplement: Supplementary file 1 — Additional file 1: Figure S1. In silico modeling of CD74-anti-CD74 scFV interaction. A Best generated models of CD74-anti-CD74 scFV interaction shown by the lowest HADDOCK score as a function of RMSD. The blue cluster was picked for further in silico mutagenesis. B Visualization of CD74-anti-CD74 scFV interaction. Red: CD74 trimer; Blue: anti-CD74 scFV. [file 40164_2023_437_MOESM1_ESM.pptx]

## Slide 1
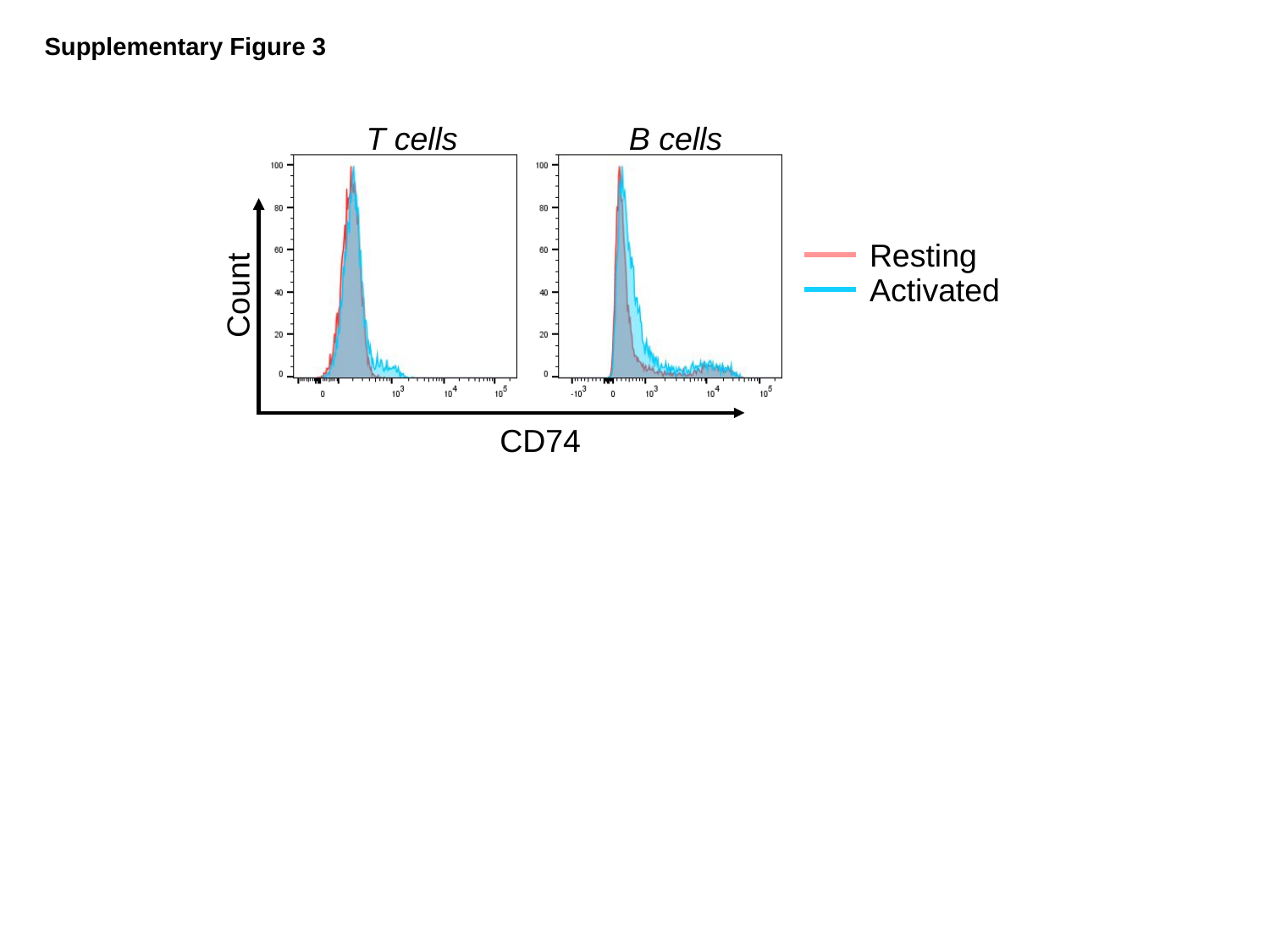

Supplementary Figure 3
T cells
B cells
Resting
Activated
CD74
Count

Supplement: Supplementary file 3 — Additional file 3: Figure S3. Expression of CD74 after activation on T cell and B cell. T cells and B cells isolated from PBMCs of 3 healthy blood donors were either untreated (red) or activated (blue) by CD3/CD28 soluble antibodies and IL-2 for T cells, and LPS (10 ng/mL)/ anti-IgM (10 µg/mL) for B cells. One representative of 3 healthy blood donors was shown. [file 40164_2023_437_MOESM3_ESM.pptx]

## Slide 1
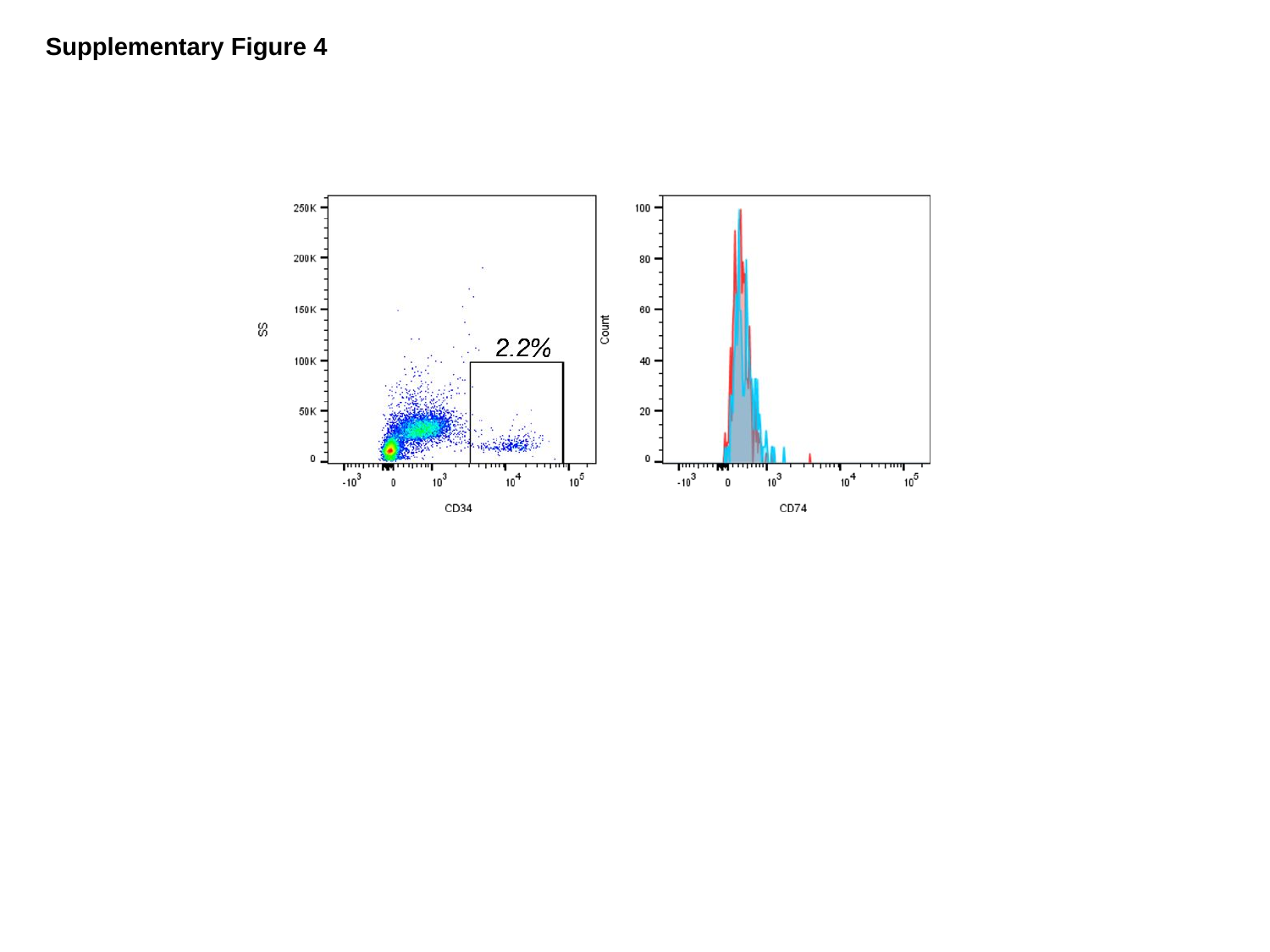

Supplementary Figure 4

Supplement: Supplementary file 4 — Additional file 4: Figure S4. Expression of CD74 on human CD34+ stem cells. Human umbilical cord blood mononuclear cells were stained with anti-CD34 and CD74 antibodies and analyzed by flow cytometry. One representative of 3 healthy cord blood donors was shown. [file 40164_2023_437_MOESM4_ESM.pptx]

## Slide 1
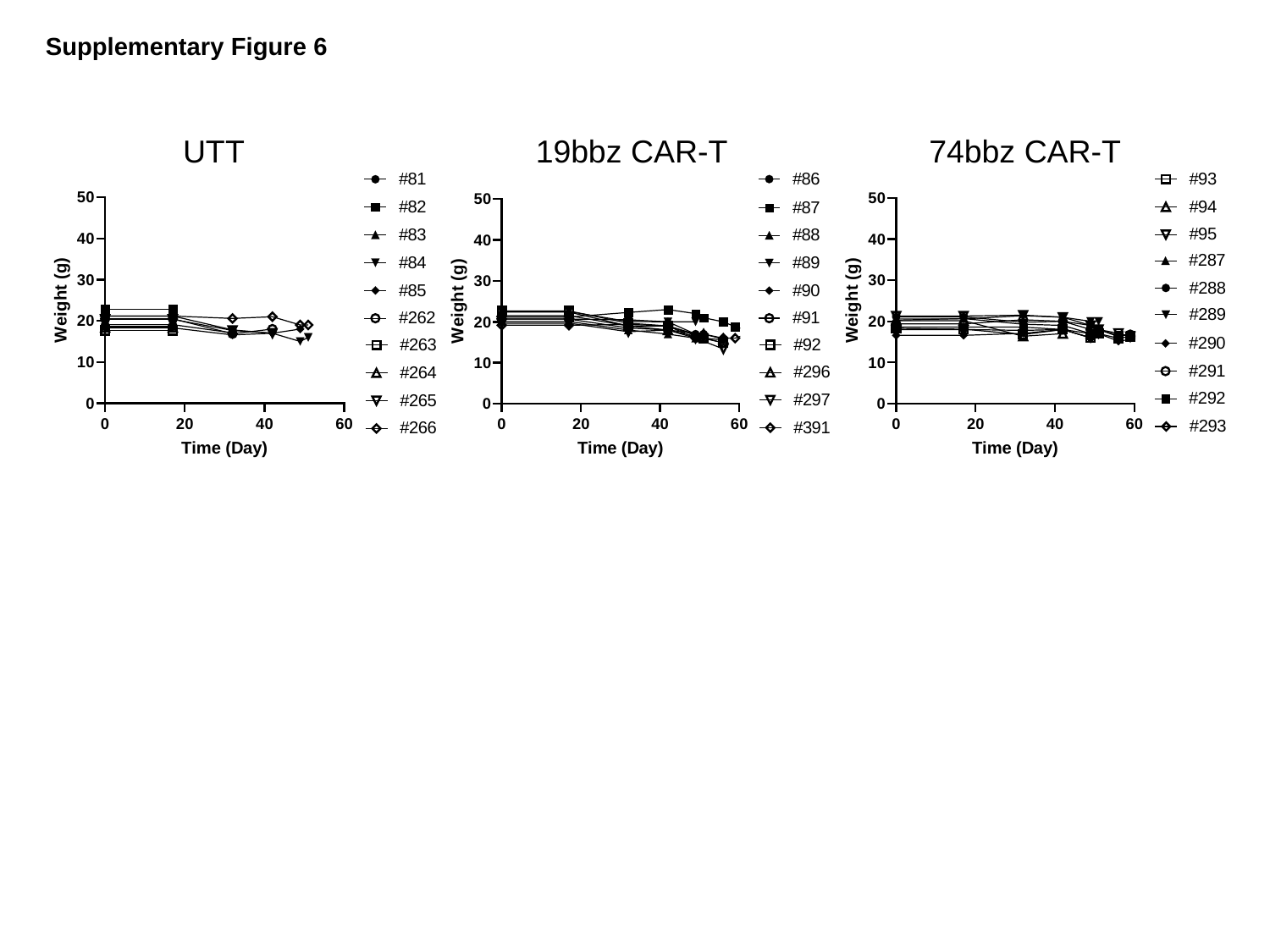

Supplementary Figure 6
UTT
19bbz CAR-T
74bbz CAR-T

Supplement: Supplementary file 6 — Additional file 6: Figure S6. No significant change in the body weights of the 74bbz CAR-T cells-treated mice was observed. The body weights of the mice treated UTT, 19bbz CAR-T and 74bbz CAR-T cells (n = 10 per group) were monitored until the mice reached ERC and plotted over time. [file 40164_2023_437_MOESM6_ESM.pptx]
