# Supplementary material for: A novel CAR-T cell product targeting CD74 is an effective therapeutic approach in preclinical mantle cell lymphoma models
Source: Exp Hematol Oncol. 2023 Sep 22;12:79. doi: 10.1186/s40164-023-00437-8 (PMC10517521; doi:10.1186/s40164-023-00437-8)
Supplement: Supplementary file 2 — Additional file 2: Figure S2. Creation of the 74bbz mutant clones. A GFP+ cells of the 74bbz mutants and parent CAR expressing Jurkat cells were sorted at the same intensity by flow cytometry. B An immunoblot of CD3ζ to show the expressing of parent, 543, 5311, 42105-74bbz clones. Endogenous CD3ζ was detected at 15 kDa while the chimeric CD3ζ on CAR was detected at 55 kDa. C CD74-ECD-Fc fusion protein was stained by Coomassie blue staining. [file 40164_2023_437_MOESM2_ESM.pptx]

## Slide 1
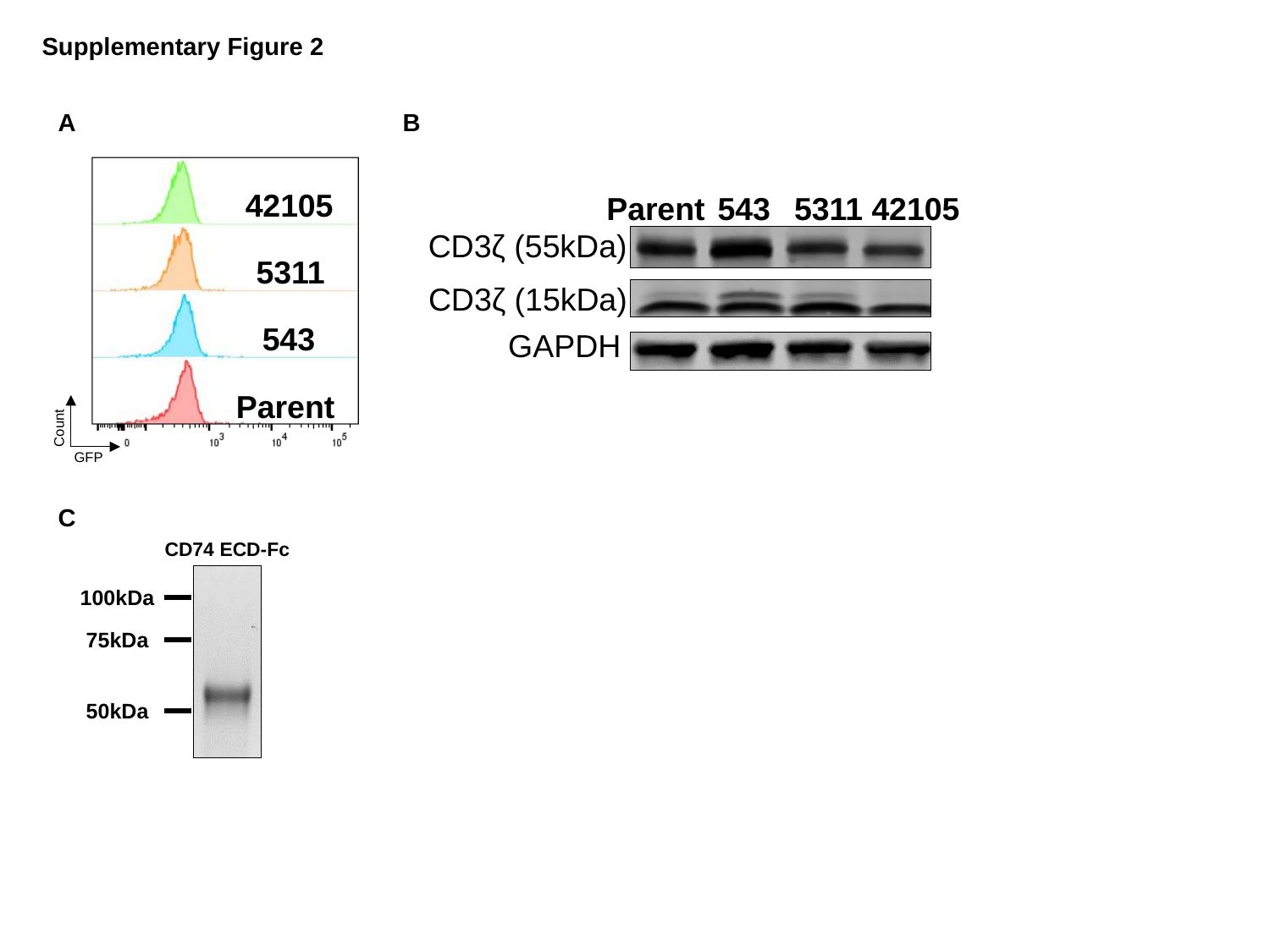

Supplementary Figure 2
A
B
42105
Parent
543
5311
42105
CD3ζ (55kDa)
CD3ζ (15kDa)
GAPDH
5311
543
Parent
Count
GFP
C
CD74 ECD-Fc
100kDa
75kDa
50kDa
