# Supplementary material for: A novel CAR-T cell product targeting CD74 is an effective therapeutic approach in preclinical mantle cell lymphoma models
Source: Exp Hematol Oncol. 2023 Sep 22;12:79. doi: 10.1186/s40164-023-00437-8 (PMC10517521; doi:10.1186/s40164-023-00437-8)
Supplement: Supplementary file 5 — Additional file 5: Figure S5. No significant depletion of circulating immune cells pre/post peak detection of 42105-74bbz CAR-T cells in a humanized mouse model. A Absolute cell numbers of B cells, monocytes, G-MDSC, M-MDSC and NK cells in humanized NSG mice on Day 3, 11 and 23 post UTT or 74bbz CAR-T engraftment. All human cells were identified by human CD45+. B: CD33−CD19+; Monocyte: LIN−CD45+CD11b+CD33+CD14+; G-MDSC: LIN−CD11b+CD33+CD14−HLA-DR−; M-MDSC: LIN−CD11b+CD33+CD14+HLA-DR−; NK: CD33−CD3−CD56+. Mice received either UTT cells (red, n = 5) and 42105-74bbz (blue, n = 7). Bars show the median cell number. B CD74 expression (blue) of B cells, monocytes, G-MDSC, M-MDSC and NK cells on Day 18 compared to isotype control (red). Data are from one mouse from the 42105-74bbz CAR-T cell treatment group. [file 40164_2023_437_MOESM5_ESM.pptx]

## Slide 1
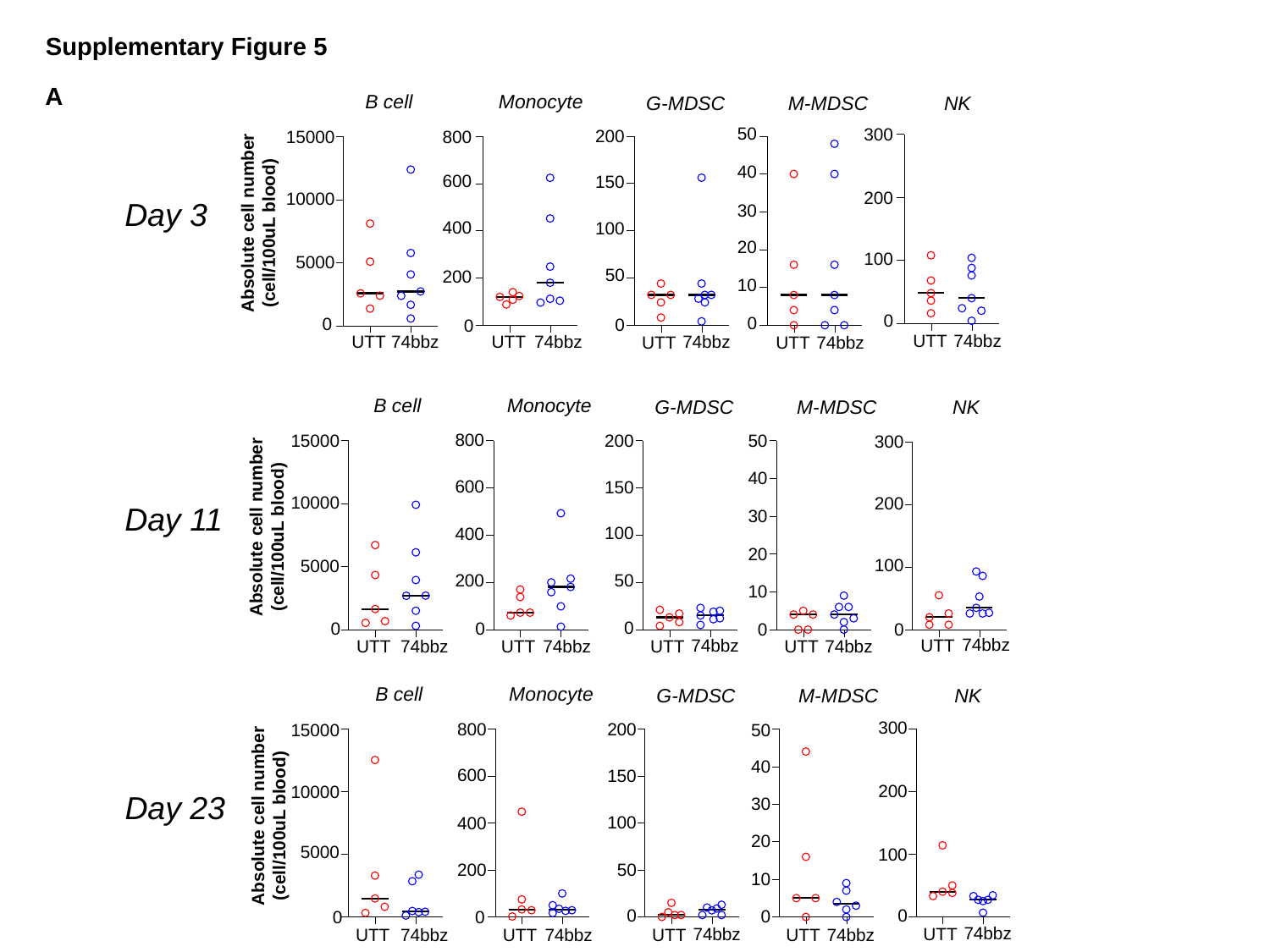

Supplementary Figure 5
A
B cell
Monocyte
G-MDSC
M-MDSC
NK
50
300
200
800
15000
40
600
150
200
10000
Day 3
30
Absolute cell number
 (cell/100uL blood)
400
100
20
100
5000
50
200
10
0
0
0
0
0
74bbz
UTT
74bbz
UTT
74bbz
UTT
74bbz
UTT
UTT
74bbz
B cell
Monocyte
G-MDSC
M-MDSC
NK
800
200
15000
50
300
40
600
150
10000
200
Day 11
30
Absolute cell number
 (cell/100uL blood)
100
400
20
100
5000
200
50
10
0
0
0
0
0
74bbz
UTT
74bbz
UTT
74bbz
UTT
74bbz
UTT
UTT
74bbz
B cell
Monocyte
G-MDSC
M-MDSC
NK
300
800
200
15000
50
40
600
150
200
10000
Day 23
30
Absolute cell number
 (cell/100uL blood)
100
400
20
5000
100
50
200
10
0
0
0
0
0
74bbz
UTT
74bbz
UTT
74bbz
UTT
74bbz
UTT
UTT
74bbz

## Slide 2
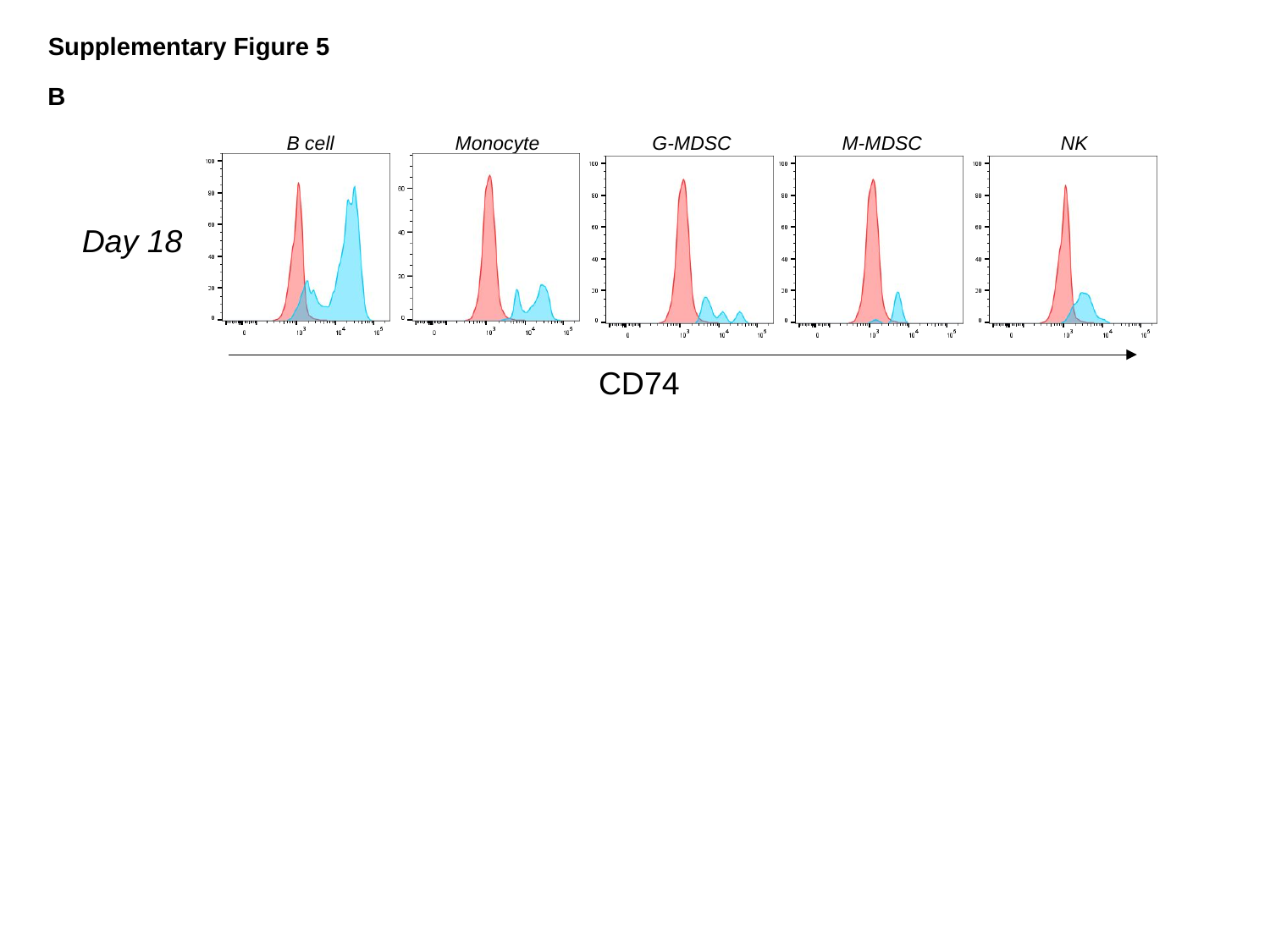

Supplementary Figure 5
B
B cell
Monocyte
G-MDSC
M-MDSC
NK
Day 18
CD74
